# Evidence for Strong Kinship Influence on the Extent of Linkage Disequilibrium in Cultivated Common Beans

**DOI:** 10.3390/genes10010005

**Published:** 2018-12-21

**Authors:** Augusto Lima Diniz, Willian Giordani, Zirlane Portugal Costa, Gabriel R. A. Margarido, Juliana Morini K. C. Perseguini, Luciana L. Benchimol-Reis, Alisson F. Chiorato, Antônio Augusto F. Garcia, Maria Lucia Carneiro Vieira

**Affiliations:** 1 Departamento de Genética, Escola Superior de Agricultura “Luiz de Queiroz”, Universidade de São Paulo, Piracicaba, São Paulo 13418-900, Brazil; augustold@usp.br (A.L.D.); giordani.willian@usp.br (W.G.); zirlane26@gmail.com (Z.P.C.); gramarga@usp.br (G.R.A.M.); augusto.garcia@usp.br (A.A.F.G.); 2 Universidade Tecnológica Federal do Paraná, Dois Vizinhos, Paraná 85660-000, Brazil; julianamorini@hotmail.com; 3 Centro de Recursos Genéticos, Instituto Agronômico de Campinas, Campinas, São Paulo 13075-630, Brazil; llasry@iac.sp.gov.br; 4 Centro de Grãos e Fibras, Instituto Agronômico de Campinas, Campinas, São Paulo 13075-630, Brazil; afchiorato@iac.sp.gov.br

**Keywords:** *Phaseolus vulgaris*, molecular polymorphism, genotyping by sequencing, population structure, GWAS

## Abstract

*Phaseolus vulgaris* is an important grain legume for human consumption. Recently, association mapping studies have been performed for the species aiming to identify loci underlying quantitative variation of traits. It is now imperative to know whether the linkage disequilibrium (LD) reflects the true association between a marker and causative loci. The aim of this study was to estimate and analyze LD on a diversity panel of common beans using ordinary $r^{2}$ and $r^{2}$ extensions which correct bias due to population structure ($r_{S}^{2}$), kinship ($r_{V}^{2}$), and both ($r_{VS}^{2}$). A total of 10,362 single nucleotide polymorphisms (SNPs) were identified by genotyping by sequencing (GBS), and polymorphisms were found to be widely distributed along the 11 chromosomes. In terms of $r^{2}$, high values of LD (over 0.8) were identified between SNPs located at opposite chromosomal ends. Estimates for $r_{V}^{2}$ were lower than those for $r_{S}^{2}$. Results for $r_{V}^{2}$ and $r_{VS}^{2}$ were similar, suggesting that kinship may also include information on population structure. Over genetic distance, LD decayed to 0.1 at a distance of 1 Mb for $r_{VS}^{2}$. Inter-chromosomal LD was also evidenced. This study showed that LD estimates decay dramatically according to the population structure, and especially the degree of kinship. Importantly, the LD estimates reported herein may influence our ability to perform association mapping studies on *P. vulgaris*.

## 1. Introduction

The major plant groups traditionally consumed for nutrition include cereals, legumes, tubers, oleaginous plants, and fruits. With some 20,000 species, the legumes (Fabaceae) are the third largest botanical family of angiosperms [1], and include the genus *Phaseolus* that is of great agricultural interest. This genus is native to the American continent, and contains 55 species, five of which are extensively farmed: *Phaseolus vulgaris* L., *Phaseolus lunatus* L., *Phaseolus coccineus* L., *Phaseolus acutifolius* A., and *Phaseolus polyanthus* Greenman [2]. The common bean (*P. vulgaris*, 2*n* = 22) is the most important species, and two clearly distinct gene pools have been identified—one Andean and the other Mesoamerican—whose molecular diversity and phenotypical characteristics, as well as their population structure and evolutionary dynamics, have been fully described in the literature [2,3,4,5,6]. Furthermore, the common bean is an important source of human nutrition. It is rich in protein and carbohydrates, and provides various nutrients essential for human health.

A number of research groups around the world have been studying the common bean in order to improve the crop, with the aim of developing more productive cultivars that are tolerant to biotic and abiotic stresses, and to boost the nutritional and technological value of the grains [7,8]. In this scenario, molecular markers have been extensively used to define the genetic architecture of agronomic traits by mapping quantitative trait loci (QTL) and estimating how many and which QTL are responsible for the phenotypic variation in the populations studied, finding their genome positions, estimating their effects and identifying interrelationships. To map QTL, it is assumed that there is linkage disequilibrium (LD), also known as gametic phase disequilibrium [9], a preferential association between loci (or haplotypes) which are transmitted as sets down through the generations [10]. In the common bean, QTL mapping has entailed linkage analysis based on endogamic populations [11,12,13,14,15,16,17,18].

Association mapping, also known as LD mapping or genome-wide association studies (GWAS), is one mapping alternative for linkage analysis, allowing better use of the genetic variation within the population and providing greater resolution in the identification of QTLs [19]. Association mapping is based on detecting genotype–phenotype associations in populations usually made up of accessions collected from the natural environment or germplasm banks. For the common bean, GWAS studies have been carried out to detect loci involved in the response to stresses caused by pathogens such as *Xanthomonas axonopodis* pv. *phaseoli* [20], *Colletotrichum lindemuthianum*, *Pseudomonas syringae* [21] and *Pseudocercospora griseola* [22], water stress [23], as well as loci involved in controlling agronomic traits [24,25,26,27] and bean cooking time [28].

To carry out GWAS experiments, it is necessary to know the genomic extent of LD and thereby define the population size and marker density, as well as the size of the LD physical window that flanks the causal polymorphism [29,30]. In this respect, a number of proposals have been put forward for assessing the statistical association among the alleles at different loci [31], the majority expressed as covariance functions or genotype correlations. The most widely used parameter is the $r^{2}$ value, which can be defined as the square of the loci correlation [32].

In regard to GWAS, only the LD produced by physical linkage is of interest. For this reason, the population structure and kinship among individuals within the population are commonly taken into account during mapping in order to avoid the detection of false associations [33]. However, the usual measurements of LD, such as $r^{2},$ do not incorporate this correction factor, resulting in biased estimates and leading to inappropriate marker density choices which impair association tests [34]. In an attempt to work around this problem, Mangin et al. [34] proposed extensions to the usual measurement ($r^{2}$) in order to control the bias introduced by population structure ($r_{S}^{2}$) or kinship ($r_{V}^{2}$) between individuals in the population under examination, as well as combining both ($r_{\mathrm{VS}}^{2}$). Plant studies that incorporate this correction have been carried out recently, for instance on eggplant (*Solanum melongena* L.) [35], barley (*Hordeum vulgare* L.) [36], cultivated beets (*Beta vulgaris* L.) [37], oat (*Avena sativa* L.) [38], pear (*Pyrus* spp.) [39], and the common grapevine (*Vitis vinifera* L.) [40].

Studies focused on estimating the LD extension based on $r^{2}$ across the *P. vulgaris* genome [4,22,41,42,43,44,45] report LD estimations as high as ~100 cM [41]. However, when accession classification into the gene pool was considered, reduced LD levels were detected, suggesting that population structure may explain the high estimate of LD between loci [46]. Valdisser et al. [47] and Resende et al. [48] also estimated the extent of LD, correcting for bias due to both population structure and kinship, and detected strong LD decay. Nevertheless, for the common bean, the relative importance of each of these factors on LD measurement is still unknown, since they were not evaluated independently in these studies.

In this scenario, the aims of the present study are to investigate the relative influence of population structure, kinship, and the combination of both to the LD estimates in a common bean diversity panel, as well as to provide a detailed examination of the intra- and inter-chromosomal LD patterns based on thousands of single nucleotide polymorphisms (SNPs).

## 2. Materials and Methods

### 2.1. Plant Material

**Figure 1:**
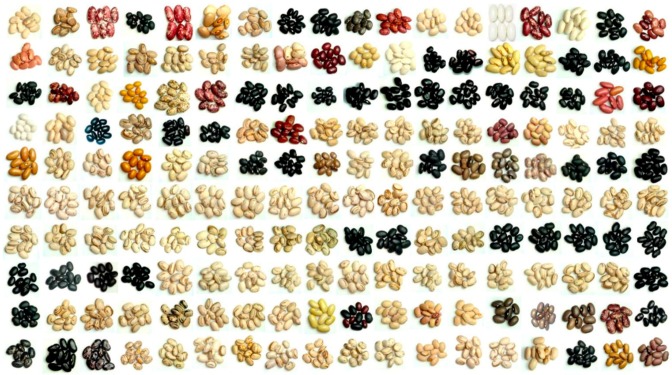
Phenotypic seed diversity encompassed by 180 accessions of a common bean panel.

This study was based on a panel of 180 common bean genotypes, as shown in Figure 1, representative of the genetic diversity of a common bean germplasm repository of 1800 accessions, deposited at the Agronomic Institute (IAC) in Campinas, Brazil. The IAC, founded in 1887, is an important research institute of São Paulo state’s Department of Agriculture and Supply. The panel included commercial cultivars from different breeding institutions, landraces, and parents of the following populations: ‘Bat 93’ × ‘Jalo EEP 558’ [49]; ‘Carioca’ × ‘Flor de Mayo’ [50]; and ‘CAL 143’ × ‘IAC UNA’, and also 14 F_10_ recombinant inbred lines (RILs) derived from the ‘CAL 143’ × ‘IAC UNA’ cross [16]. In addition to 87 inbred lines from the IAC breeding program, the diversity panel studied herein includes 62 common bean lines from the International Center for Tropical Agriculture (CIAT), 12 from the Brazilian Agricultural Research Corporation (Embrapa), as well as nine Brazilian landraces and ten cultivars [51]. Phenotypically, this panel contains variability in (*i*) grain morphology, (*ii*) resistance to biotic factors such as pests and diseases, (*iii*) tolerance to abiotic factors such as drought, and (*iv*) the micronutrient composition of grains [51]. The 180 accessions were classified according to: (*i*) institution of origin (62 from CIAT, 87 from IAC, 12 from Embrapa, 1 from each of EPAGRI (Santa Catarina State Rural Extension and Agricultural Research Enterprise) and FEPAGRO (State Foundation for Agricultural Research), 2 from FT Sementes, 4 from IAPAR (Agronomic Institute of Parana), 1 from UEM (State University of Maringa), 1 from UFLA (Federal University of Lavras), and 9 landraces); (*ii*) type of phaseolin (27 T-type and 153 S-type, of Andean and Mesoamerican origin, respectively), following the methodology proposed by Kami et al. [52]; (*iii*) grain size (42 small, 113 medium, and 25 large), and (*iv*) commercial group (1 ‘Amendoim’, 45 ‘Black’, 80 ‘Carioca’, 8 ‘Creme’, 4 ‘Jalo’, 10 ‘Mottled’, 8 ‘Mulatinho’, 5 ‘Pink’, 2 ‘Pinto Beans’, 6 ‘Red’, 6 ‘White’, 4 ‘Yellow’, and 1 ‘Zebra’), as shown in Table S1.

### 2.2. DNA Extraction, Genotyping by Sequencing Library Preparation, Sequencing, and SNP Calling

Total genomic DNA from the IAC panel genotypes (*n* = 180) was extracted from young leaves collected from 10 plants per accession using the DNeasy^®^ Plant Mini Kit (Qiagen, Venlo, Netherlands) according to the manufacturer’s instructions. DNA concentration was assessed using spectrophotometry (NanoDrop 2000, Thermo Scientific, Waltham, MA, USA) and agarose gel electrophoresis (0.8% *w*/*v*). Sample DNA intensities were compared to a DNA quantitation standard after staining with SYBR SAFE^®^ (Invitrogen, Carlsbad, CA, USA). To check DNA integrity, 500 ng from 20 randomly selected genotypes was subjected to restriction digestion using *Hind*III (New England BioLabs^®^, Ipswich, MA, USA) according to the manufacturer’s instructions, followed by electrophoresis on SYBR SAFE^®^ (Invitrogen) stained agarose gel (0.8% *w*/*v*).

Two genotyping by sequencing (GBS) libraries were constructed in 95-plex. For each library, a single random blank well was included for quality control to ensure that libraries were not switched during construction, sequencing, and analysis. Genomic DNA was co-digested with the restriction enzyme *Ape*KI (5′ CWGC 3′) and barcoded adapters were ligated to individual samples. The samples were pooled by plate into libraries and amplified by polymerase chain reaction. Detailed protocols can be found in Elshire et al. [53]. Each library was single-end sequenced to 100 bp in a single lane of HiSeq 2500 (Illumina, San Diego, CA, USA.). These procedures were carried out at the Genomic Diversity Facility, Institute of Biotechnology, Cornell University, USA.

Sequences from two *Ape*KI-GBS libraries from the IAC panel can be downloaded from GenBank BioSample SAMN05513252 and SAMN05513251, both included in BioProject PRJNA336556.

The TASSEL-GBS bioinformatics pipeline [54], designed for efficiently processing raw GBS sequence data into SNP genotypes, was used in the present study. Sequences from an inbred landrace line of *P. vulgaris* (G19833) were set as the reference genome [55]. Initial filtering was based on the following settings: (*i*) minor allele frequency (MAF) ≥ 0.01 and (*ii*) minimum coefficient of inbreeding 0.9.

### 2.3. Filtering and Imputing Genotyping by Sequencing SNP Calls

Only SNPs in the assembled chromosomal pseudomolecules of *P. vulgaris* were selected. Exploratory analyses were conducted in order to verify the proportion of missing and heterozygous data for each SNP data set. Because *P. vulgaris* is predominantly autogamous, the occurrence of heterozygotes is negligible [12], as shown in Figure S1. We therefore assumed that these cases were possible sequencing errors and treated them as missing data. A 10% threshold for missing data was set prior to imputing. The method proposed by Roberts et al. [56] was used for imputing missing/unknown SNP data. This method is particularly suitable for inferring missing genotype information in large sets of SNPs from inbred lines, based on the information at adjacent loci, i.e., the existence of LD between loci (haplotypes). Accuracy is increased based on the prediction of known genotypes and the method is widely used for autogamous species. Initially, to determine the optimal number of loci to be set as the imputing window size, we evaluated five to 150 loci and selected the one with the highest accuracy. The window size was set, and the NPUTE package [56] was used to perform data imputation chromosome-by-chromosome. Then a 5% MAF threshold was set and the remaining SNPs were used for determining population structure, kinship, and LD analysis.

### 2.4. Population Structure, Kinship, and Linkage Disequilibrium Investigation

Principal component analysis (PCA) was applied to investigate population structure, via the ‘prcomp’ function implemented in the R statistical package [57], and a Tracy–Widom statistic test was used to define the number of significant principal components (PCs) [58,59]. The criteria for determining the number of PCs used as a population structure (S) matrix for LD measurements were based on (*i*) the proportion of variance explained by each PC and (*ii*) graphic visual inspection of the dispersion of PC scores. Therefore, we used the first four PC values as the ‘S’ matrix. In addition, nucleotide diversity π [60] was estimated using MEGA5 [61].

A relatedness matrix was estimated from genotype data using the simple matching coefficient, extended to include loci that are identical by state but not by descent [62], according to Equation (1):

(1) $$\frac{S-S_{min}}{1-S_{min}}$$

where S is the matrix of simple matching coefficients corrected by the minimum of observed simple matching coefficients, $S_{min}$. The ‘kin’ function in the synbreed package of the R platform [63] was used with argument ret = “sm−smin”.

Linkage disequilibrium, the non-random association of alleles at two different loci, was estimated by squared allele-frequency correlations using R package “LDcorSV” [34]. Four LD estimates were calculated: conventional $r^{2}$ based only on genotype data, $r^{2}$ correcting for population structure bias ($r_{S}^{2}$), $r^{2}$ taking account of kinship ($r_{V}^{2}$), and $r^{2}$ with both population structure and kinship included ($r_{VS}^{2}$). The p-values for each test were obtained by applying Fisher’s exact test run using the ‘LD’ function implemented in R package “genetics” [64]. In addition, the false discovery rate (FDR) was controlled by selecting tests with significance of 5% [65].

LD decay over genetic distance was investigated by plotting pairwise LD values against the distance between loci on the same chromosome. It was modelled by matching a modified recombination-drift model [66], including a low level of mutation and adjustment for sample size, using the Hill and Weir [67] expectation of $r^{2}$ between adjacent sites, according to Equation (2):

(2) $$E\left(r^{2}\right)=\left[\frac{10+C}{\left(2+C\right)\left(11+C\right)}\right]\left[1+\frac{\left(3+C\right)\left(12+12C+C^{2}\right)}{n\left(2+C\right)\left(11+C\right)}\right]$$

where n is the sample size, and C, the parameter to be estimated, represents the product of the population recombination parameter $\rho =4N_{e}r$ and the distance in base pairs. Finally, heatmaps were produced based on pairwise LD measurements for all marker pairs within each chromosome in order to visualize intra-chromosomal LD patterns. Additionally, inter-chromosomal LD was investigated based on $r_{VS}^{2}$ ≥ 0.7, comparing SNPs located on different chromosomes.

## 3. Results

### 3.1. Single Nucleotide Polymorphism Calling and Imputation

After sequencing the two GBS libraries from the IAC panel, a total of 428,404,611 reads were obtained, of which 399,296,160 (93.2%) were of high quality, and 3,018,395 tags were identified. Regarding alignment, 1,678,051 (55.6%) aligned to single positions on the reference genome of *P. vulgaris* and 163,651 (5.4%) aligned to multiple regions. Finally, 83,364 SNPs were identified on chromosomal pseudomolecules and 684 on scaffold sequences from the reference genome.

As the frequency of heterozygous genotypes was practically null, as shown in Figure S1, we assumed these cases to be possible sequencing errors and therefore treated them as missing data. The proportion of missing data was less than 50% for over 70% of the loci identified.

In the process of replacing missing data, the number of loci per window size varied from 10 (Pv09) to 22 (Pv07), as shown in Table S2. In terms of MAF distribution, imputation did not make any significant difference to the final data set, since a very conservative threshold for missing data was applied, as shown in Figure S2.

Finally, after filtering, a total of 10,362 SNPs were identified, as shown in Table 1. Polymorphisms were widely distributed along the 11 chromosomes of *P. vulgaris*, although not uniformly (a higher SNP density was found near the chromosome ends). The chromosome position, alleles, and MAFs of filtered SNPs are given in Table S3.

### 3.2. Population Structure and Kinship

**Figure 2:**
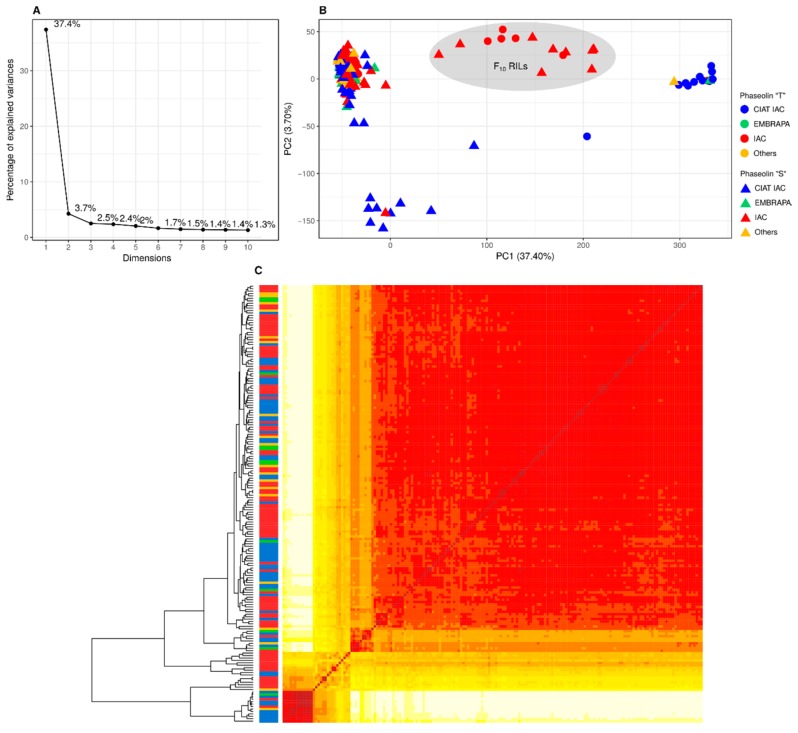
Population structure inferred by principal component analysis (PCA) (**A** and **B**) and the dendrogram and the heatmap (**C**) of a kinship matrix estimated by the simple matching coefficient, extended to account for loci that are identical by state but not identical by descent, based on 10,362 single nucleotide polymorphism (SNP) markers (minor allele frequency > 5%), among 180 genotypes from the IAC diversity panel. The colored shapes (**B**) were classified in two groups in accordance with the type of phaseolin: “T” (circle) and “S” (triangle), which have Andean and Mesoamerican origin, respectively. The colors shown in B and the scale between the dendrogram and the heatmap (**C**) corresponds to the breeding institution.

According to PCA, the first PC accounted for the majority (37.4%) of genetic variation, as shown in Figure 2A, and was generally consistent with prior gene pool classification—Andean vs. Mesoamerican, as shown in Figure 2B. The second PC accounted for 4.3% of the variation and revealed substructuring among the Mesoamerican accessions, in which nine accessions from CIAT were clearly differentiated from the others. Combining the first two PCs, four groups were formed, the largest of which includes 144 phaseolin type “S” accessions, with no relationship between the grouping and the institution of origin. This group also includes all IAC accessions with typically ‘carioca’ grains (51), and most of the accessions with typically ‘black’ grains (18), both of considerable commercial interest in Brazil.

A further two separate groups were, for the most part, accessions originating from CIAT. One of them includes only “S” accessions, and the other consists mainly (9 accessions) of “T” accessions of Andean origin, with the large grains typical of this gene pool. In addition, nucleotide diversity analysis, as shown in Table 2, suggests that the CIAT collection (π = 0.309) is more diverse than IAC, Embrapa, and accessions from other institutions.

Finally, there was a separate group including the 14 F_10_ recombinant inbred lines (RILs) produced by bi-parental crossing of the accessions ‘CAL 143’ (Andean) and ‘IAC Una’ (Mesoamerican) [16,17,68,69]. Estimating the degree of kinship once again revealed that there was a tendency for accessions from the same breeding institution to cluster together, as shown in Figure 2C.

### 3.3. Linkage Disequilibrium

**Figure 3:**
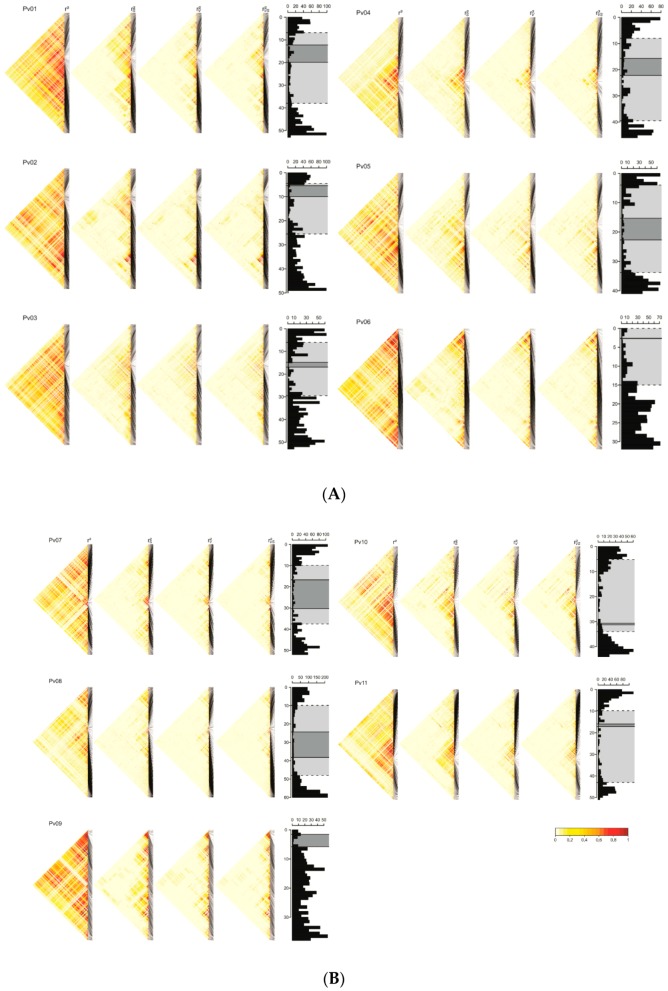
Linkage disequilibrium (LD) patterns in cultivated *Phaseolus vulgaris* from the IAC diversity panel. Histograms indicate SNP density along the chromosome: (**A**) chromosomes Pv01 to Pv06; (**B**) chromosomes Pv07 to Pv09. Ordinate and abscissa correspond to the loci position (Mb) and number of SNPs, respectively. The areas delimited by continuous and dashed lines correspond to centromeric and pericentromeric regions, respectively. LD heatmaps are shown for $r^{2}$ measurements and extensions correcting bias from population structure ($r_{S}^{2}$), kinship ($r_{V}^{2}$), and both combined ($r_{VS}^{2}$). The degree of LD is indicated by colors from light yellow (no LD) to red (strong LD).

Analyzing the $r^{2}$ values calculated for SNP pairs in the same chromosome, there is a tendency for average LD values to decrease as the distance between loci increases, as shown in Figure 3. However, values of $r^{2}$ > 0.8 were detected between pairs of SNPs at a distance of 10 Mb and between SNPs at opposite ends of the chromosome.

In contrast, distinct LD patterns were obtained when the measurements that control the bias introduced by population structure ($r_{S}^{2}$), kinship ($r_{V}^{2}$), and both combined ($r_{VS}^{2}$) were taken into account. In all cases, there was a drastic decrease in the LD estimates, although they remained high between closely linked loci. Compared to $r_{S}^{2}$, the estimates of $r_{V}^{2}$ were lower overall, showing that the bias ascribed to kinship was higher compared to that ascribed to population structure. Furthermore, the results obtained for $r_{V}^{2}$ and $r_{VS}^{2}$ were very similar, suggesting that kinship includes information on population structure. Regardless of the correction applied, the largest blocks of loci with high LD were detected in the centromeric and pericentromeric regions, in which recombination is inhibited. However, there were also blocks in the distal region of the long arm of chromosomes Pv06 and Pv09.

**Figure 4:**
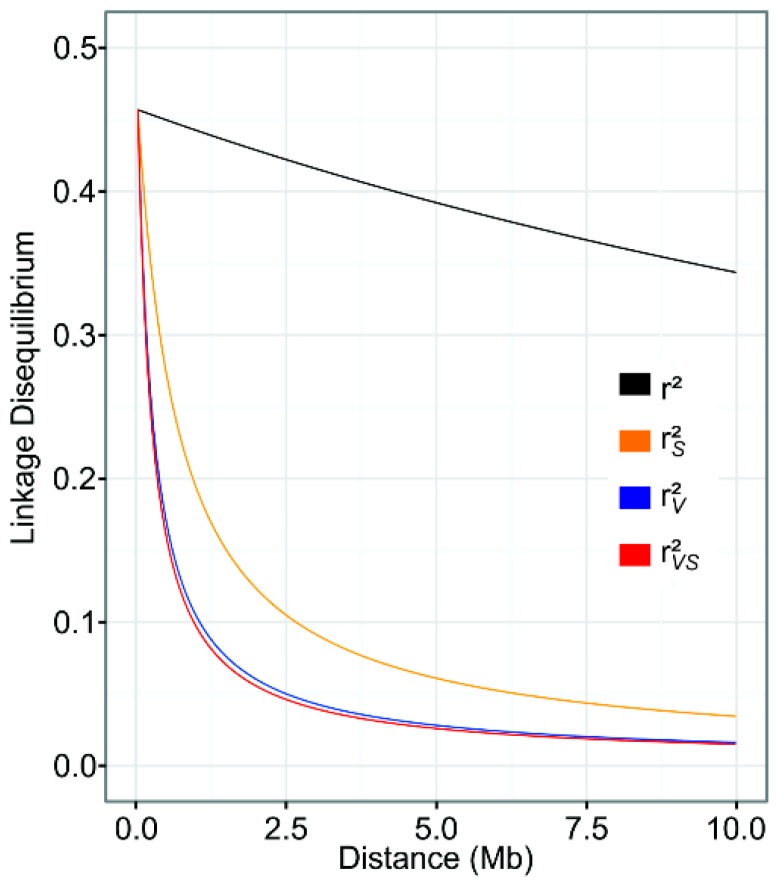
Linkage disequilibrium (LD) decay determined by four LD measurements against distance between SNPs within the chromosome, adjusted by the mutation model.

When adjusted to fit the model proposed by Hill and Weir [67] regarding estimated LD ($r^{2}$) as a function of distance, there was subtle decay observed. However, when the biases ascribed both to population structure and kinship were taken into account, LD decayed to 0.1 at distances of around 1 Mb, and again very similar results were obtained for $r_{V}^{2}$ and $r_{VS}^{2}$, as shown in Figure 4.

**Figure 5:**
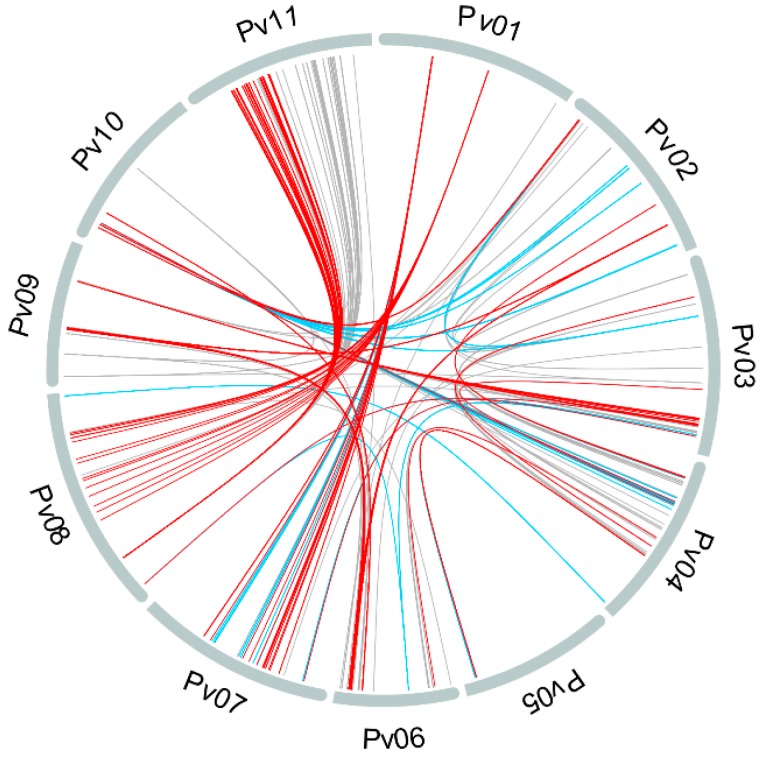
Inter-chromosomal linkage disequilibrium (LD) in *Phaseolus vulgaris*. Gray, blue, and red lines connecting chromosomes correspond to LD estimates between SNP pairs in which $r_{VS}^{2}$ ≥ 0.7, 0.8, and 0.9, respectively.

Even using $r_{VS}^{2}$, high LD values (≥0.7) were detected between 671 SNP pairs located in different chromosomes, revealing inter-chromosomal LD patterns, as shown in Figure 5. We found values of $r_{VS}^{2}$ ≥ 0.9 between two loci from Pv01, with 20 and 16 others distributed respectively on Pv07 and Pv08. Furthermore, preferential associations between a locus on Pv08 with loci on the pericentromeric region of Pv11 were detected.

## 4. Discussion

In this study, we examined intra- and inter-chromosomal LD patterns in 180 genotypes of cultivated beans from a diversity panel based on information on 10,362 high-quality SNPs. Random sequencing coverage of genomic regions from different samples and mutations at restriction sites resulted in missing data, due to the GBS technique [53]. Nevertheless, the percentage of missing data was <50% for the majority (>70%) of the SNPs analyzed herein. In addition, since a very conservative threshold for missing data was applied, the imputation step did not result in significant differences in the final data set. We performed the Pearson’s correlation estimation between the two data sets (before and after imputation), which are over 98% correlated. The distribution of SNPs was not uniform along the chromosomes. This is due to the reduction in genome complexity when libraries are built by enzymatic hydrolysis of the DNA using *Ape*KI, whose activity is inhibited in methylated regions. As expected, centromeric and pericentromeric regions, identified in silico by gene density and repetitive elements [55], exhibited lower SNP density.

### 4.1. Population Structure and Kinship

In terms of PCA, the first two principal components accounted respectively for 37.4% and 4.3% of genetic variation, as shown in Figure 2A. This allowed us to classify the gene pools coherently as Andean vs. Mesoamerican, with the exception of 14 F_10_ RILs, as shown in Figure 2B, which were inter-pool hybrids produced by crossing the accessions ‘CAL 143’ (Andean) and ‘IAC Una’ (Mesoamerican). Furthermore, PCA revealed Mesoamerican accession substructuring, which corroborated earlier reports indicating that the Mesoamerican pool contains more diversity than the Andean pool [4,5,70]. Within the Mesoamerican pool, the 144 phaseolin type “S” accessions consisted of plants with medium-sized or small seeds—a typical Mesoamerican characteristic—lending weight to a classification based on the phaseolin protein. In addition, the remaining nine Mesoamerican CIAT accessions were distinguished from the others, indicating higher diversity of the CIAT collection compared those of the other institutions, as confirmed by the nucleotide diversity analysis, as shown in Table 2.

The IAC diversity panel contained accessions from different breeding institutions. Although not as strong due to significant germplasm exchange between breeding programs, kinship estimation revealed a bias for grouping accessions according to these institutions, as shown in Figure 2C. Since a select group of parents is preferentially used to produce commercial varieties at breeding institutions, the genetic base can sometimes be narrow, and a significant degree of kinship is to be expected among genotypes from the same breeding institution.

### 4.2. Linkage Disequilibrium

Estimates of LD varied from one chromosomal region to another and were higher in pericentromeric regions. Similarly, in the soybean, it has been reported that LD is negatively correlated (*r* = −0.47) with recombination rates in these regions [71]. In our study, estimates based on the ordinary measurement ($r^{2}$) indicated that LD remains high even between loci at opposite ends of the chromosome. Recently, very similar results were obtained for the common bean, and Valdisser et al. [47], studying a core collection genotyped by the DArTseq high-density SNP approach, reported that $r^{2}$ does not reflect the decay of LD over physical position, and Blair et al. [43] also reported that LD measured by $r^{2}$ decays slowly as a function of genetic and physical distances.

In many cases, $r^{2}$ clearly overestimates LD along a chromosome, which can be explained by various factors, such as artificial selection, population structure, and the occurrence of inbreeding or kinship between the genotypes within a population [34]. When studying LD in *P. vulgaris*, some authors group the genotypes according to the population structure prior to $r^{2}$ estimation and compute this measurement independently for each gene pool. For instance, Rossi et al. [46] and Valdisser et al. [47] evaluated the effect of population structure on LD and found differences between Andean and Mesoamerican gene pools, indicating that population structure is a significant factor influencing the magnitude of LD in common bean. These studies reported slower LD decay for the Andean population compared to the Mesoamerican. Similarly, Blair et al. [43] demonstrated clear gene pool differences, with the majority of LD explained by population structure. In addition, we found that the IAC panel revealed substructuration within gene pools, especially for Mesoamerican genotypes, where LD was stronger and decayed more slowly. These different substructures below gene pool level may also influence LD, and as shown by Kwak and Gepts [4], LD estimates in further subdivisions are not accurate, due to small sample size populations, a limitation that can be overcome by applying the appropriate correction to $r^{2}$.

In order to account for population structure and kinship, we applied three different corrections to $r^{2}$, as proposed by Mangin et al. [34]. Our results show that the $r_{S}^{2}$, $r_{V}^{2}$, and $r_{VS}^{2}$ measurements may help to tackle the problem of the bias introduced by linkage disequilibrium estimates, especially those resulting from the population structure or relatedness of individuals. The IAC panel consists of cultivated accessions and includes lines derived from advanced breeding stages, thus presenting higher LD than expected for wild populations. This kind of comparative analysis has already been conducted; for instance, Rossi et al. [46] found a mean $r^{2}$ of 0.08 and 0.18 in wild and domesticated beans, respectively.

As expected, when we analyzed the highly structured IAC panel, the $r_{S}^{2}$ estimate improved the correction of $r^{2}$ bias, although this was not sufficient to eliminate all the bias due to relatedness. Our results show further advantages for using $r_{V}^{2}$ to account for the kinship bias, indicating that the bias ascribed to kinship is higher than that ascribed to population structure. Furthermore, the similar results obtained for $r_{V}^{2}$ and $r_{VS}^{2}$ suggest that population structure is already taken into account in kinship estimation, especially since accessions from the same pool are closely related. Similar results have already been reported for other species, such as cultivated beets (*Beta vulgaris* L.) [37], oat [38], and grapevine (*Vitis vinifera* L) [40].

Interestingly, even working on a highly structured panel, $r_{V}^{2}$ may have advantages over $r_{S}^{2}$, revealing that in some cases modeling genetic relationships by the kinship matrix may be enough to correct the LD bias. Furthermore, our findings suggest that the degree of kinship among individuals in the population under study is worth of special attention. In panels substantially consisting of improved lines, such as those used herein, the bias induced by kinship is stronger than that of population structure itself.

Even using parameters that, in theory, remove most of the bias from $r^{2}$ values, we found that LD in *P. vulgaris* decayed to 0.1 at a distance of 1 Mb between loci, indicating fairly widespread LD within the species, in contrast to that observed in cultivars of soybean and rice. In these cases, the authors reported decays at a distance between loci of 133 kb (*Glycine max* [71]), 123 kb (*Oryza sativa* Indica [72]), and 167 kb (*Oryza sativa* Japonica [72]). For the common bean, the decay reported herein is slower, even compared to other studies in which structure and kinship bias was corrected, and reporting a decay to 0.1 at around 400 Kb [47] and 700 Kb [48]. Nonetheless, the fit of the LD decay curve should be interpreted with caution, since there are regions in which LD decays rapidly, showing that there are specific patterns in the common bean genome. The existence of large LD blocks in the distal region of the long arm of chromosomes Pv06 and Pv09 corroborates the results obtained by Bhakta et al. [73]. These authors determined the recombination rate along arms of individual bean chromosomes. For instance, both arms of chromosome 6 are dominated by a block of rDNA repeats that interferes with the recombination activity.

Inter-chromosomal LD was recently reported by Campa et al. [44] in the common bean, but with different patterns compared to those we found for the IAC panel. The fact that inter-chromosomal LD has been observed, even taking into account the correction for population structure and kinship bias, implies that the breeding process may contribute to LD magnitude. According to Perseguini et al. [22], population mating systems could heavily influence LD patterns in *P. vulgaris*, as well as epistatic effects, which have been reported to control seed yield and other agronomic traits in an Andean × Mesoamerican cross [74]. Furthermore, diverse LD patterns may also be associated with domestication events for the Andean and Mesoamerican gene pools, which selected indirectly for different chromosomal regions [44,55].

The GWAS method is based on the detection of genotype–phenotype associations in populations consisting of genotypes originating from natural populations and germplasm bank accessions. These populations often span many generations and it is assumed that multiple recombination events have occurred, reducing the extent of genomic regions affected by LD. The results of this study show that the marker density required for reasonable coverage of the genome depends on the particular features of the common bean genome and the chromosomal context. Additional studies to explore patterns of specific genomic segments with reduced LD could be conducted to carry out LD mapping based on a candidate gene approach, for example, as used to dissect the genetic architecture of plant shape in rice [75]. In addition, characterization of loci on chromosomes with high LD and regions with extensive LD may disclose important loci related to signatures of domestication and artificial selection used in breeding programs. Finally, this kind of approach could provide a more complete picture of the magnitude and structure of LD in the common bean.

Our findings have fundamental implications for the development of association mapping in the common bean. In particular, it is already evident that the careful evaluation of population structure is a key element. Furthermore, our findings suggest that the degree of kinship among individuals in the population under study is also worthy of special attention. In panels substantially consisting of improved lines, such as those used herein, the bias induced by kinship may be stronger than that of population structure itself.

Finally, we would stress the need to take account of one-off LD patterns in genomic regions in which studies have detected significant associations with phenotypic traits. Based on this analysis, it will be possible to outline strategies for exploiting these regions, with the aim of identifying candidate genes involved in trait inheritance.

## 5. Conclusions

This study shows that LD estimates decay dramatically if population structure is taken into account, and especially the degree of kinship among accessions of cultivated common beans on the basis of information on thousands of SNPs. LD measurements vary from one chromosomal region to another and, as expected, are higher in pericentromeric regions. We also found evidence of LD between inter-chromosome loci, suggesting that the breeding process and the species crossing system may have contributed to LD magnitude, since the bias due to population structure and kinship was corrected for. Importantly, the LD estimates herein may influence our ability to localize important genes on the basis of association mapping studies in *P. vulgaris*.

## Figures and Tables

**Table 1:** Number of single nucleotide polymorphisms (SNPs) per chromosome of *Phaseolus vulgaris* generated by *Ape*KI-GBS (genotyping by sequencing) technology in the IAC (Agronomic Institute) diversity panel.

			Number of SNPs	
Chromosome	Physical Length (Mb) ^1^	Number of Genes ^1^	MD ≤ 0.10	MAF ≥ 0.05
Pv01	52.18	2116	2100	993
Pv02	49.03	2695	2678	1261
Pv03	52.21	2294	2407	1063
Pv04	45.79	1035	1378	748
Pv05	40.23	1349	1335	695
Pv06	31.97	1649	1635	841
Pv07	51.69	2146	2133	1082
Pv08	59.63	2067	2453	1188
Pv09	37.39	2134	1935	869
Pv10	43.21	1020	1379	680
Pv11	50.20	1274	1848	942
Total	−	19,779	21,281	10,362

MD: missing data; MAF: minor allele frequency. ^1^ According to the *Phaseolus* *vulgaris* reference genome [55].

**Table 2:** Nucleotide diversity in a common bean diversity panel, based on SNPs generated by *Ape*KI-genotyping by sequencing (GBS) technology. CIAT: International Center for Tropical Agriculture.

Institution of Origin	N	S	π
IAC	87	10,354	0.256
CIAT	62	10,346	0.309
Embrapa	12	9632	0.272
Other	19	9984	0.251
Total	180	10,362	0.277

**N**: number of accessions; **S**: number of SNPs, and π: nucleotide diversity [60].

## Supplementary Materials

The following are available online at http://www.mdpi.com/2073-4425/10/1/5/s1. Figure S1: Exploratory analysis of raw data sets of SNPs generated by *Ape*KI-GBS technology from the IAC panel (82,680 SNPs); Figure S2: Minor allele frequency distribution of SNPs before (pink) and after (blue) imputation, from the IAC panel; Table S1: IAC diversity panel: classification according to institution of origin, type of phaseolin, gene pool, grain size, and commercial group; Table S2: Imputation analysis parameters: window size and accuracy of SNPs generated by *Ape*KI-GBS technology in the IAC diversity panel; Table S3: Chromosome position, alleles, minor allele frequency (MAF), and genotypes of 10,362 filtered SNPs generated by *Ape*KI-GBS technology in the IAC diversity panel.
