# Supplementary figures and images for: Evidence for Strong Kinship Influence on the Extent of Linkage Disequilibrium in Cultivated Common Beans
Source: Genes (Basel). 2018 Dec 21;10(1):5. doi: 10.3390/genes10010005 (PMC6356217; doi:10.3390/genes10010005)

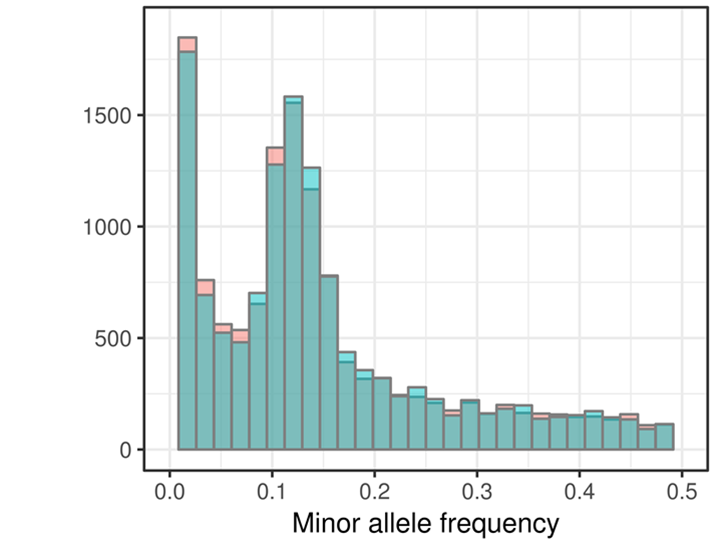

Supplement: Supplementary file 1 [file genes-10-00005-s001.zip › Figure_S2.tif]
